# From diabetes to dentistry: metformin targets mitochondrial-immune crosstalk to restore periodontal homeostasis

**DOI:** 10.1186/s12967-026-07772-4

**Published:** 2026-02-20

**Authors:** Mingjie Wu, Feng Wang, Yuan Zhang, Aili Xing, Zhongrui Li, Xiangxiang Lu, Yuwen Lai, Bin Zhao, Bin Sun

**Affiliations:** 1 Oral and Maxillofacial Surgery, Hospital of Stomatology, Jilin University, Changchun, 130021 China; 2 Periodontics, Hospital of Stomatology, Jilin University, Changchun, 130021 China; 3 Jilin Provincial Key Laboratory of Oral Biomedical Engineering, Changchun, 130021 China

**Keywords:** Metformin, Periodontitis, Autophagy, Pyroptosis, Local drug delivery, AMPK signaling

## Abstract

**Background:**

Metformin (MET), a first-line antidiabetic agent, exhibits significant therapeutic potential for periodontitis management due to its multifaceted pharmacological actions.

**Main body:**

This review synthesizes current evidence on MET’s antimicrobial, anti-inflammatory, and osteogenic properties in the context of periodontitis. Mechanistically, MET primarily targets mitochondrial Complex I, inhibiting ATP production and activating AMPK. This AMPK activation enhances microtubule dynamics via CLIP170 phosphorylation, thereby boosting the bactericidal capacity of neutrophils and macrophages. Furthermore, MET suppresses mTOR signaling, which promotes M2 macrophage polarization, regulates autophagic flux, and inhibits NLRP3-mediated pyroptosis, collectively mitigating periodontal inflammation and tissue damage. In the realm of bone regeneration, MET upregulates key osteogenic markers and improves alveolar bone volume in preclinical models. Recent translational advances are focusing on the development of localized MET delivery systems, such as hydrogels, electrospun fibers, and MET-functionalized scaffolds, to enhance therapeutic efficacy at the site of disease.

**Conclusions:**

MET represents a promising multifunctional agent for periodontitis therapy, effectively bridging antimicrobial and regenerative therapeutic strategies. However, several challenges remain, including the need to define optimal concentrations for osteogenesis and to obtain robust clinical validation for novel delivery systems. Future research should prioritize systematic dose optimization, mechanistic exploration beyond the canonical AMPK pathway, and the execution of large-scale clinical trials to translate these promising preclinical findings into established clinical practice.

Periodontitis represents a multifactorial inflammatory condition characterized by complex host-microbe interactions. While oral microbial biofilm serves as the primary etiological agent, host susceptibility plays a pivotal role in determining disease progression and severity [1]. The pathogenesis of periodontitis involves a complex interplay, including subgingival microbial dysbiosis, host genetic predisposition, and the dualistic nature of host immune responses. The clinical presentation progresses through a series of characteristic stages, like gingival inflammation, periodontal pocket formation, alveolar bone resorption, and tooth mobility. Metformin (MET), a biguanide derivative with pleiotropic properties, has established itself as the cornerstone of type 2 diabetes mellitus(T2DM) management [2]. Beyond its primary hypoglycemic action, accumulating evidence has revealed its therapeutic potential in addressing metabolic syndrome components, including obesity [3], dyslipidemia, and cardiovascular disease [4, 5]. Recent advances in understanding MET’s molecular mechanisms and tissue-specific actions have expanded its therapeutic repertoire, encompassing diverse pathological conditions, including COVID-19 [6]. Particularly noteworthy is the growing body of research investigating MET’s role in chronic periodontitis management, especially in the context of diabetes-associated periodontitis. MET demonstrates a tripartite therapeutic mechanism in periodontitis, encompassing antimicrobial activity against periodontal pathogens, modulation of host inflammatory cascades, and stimulation of periodontal tissue regeneration. This comprehensive review critically examines current evidence regarding MET’s therapeutic potential in periodontitis, with particular emphasis on its mechanisms of action (Figs. 1 and 6), aiming to provide a scientific foundation for its potential clinical applications in periodontal therapy.

**Fig. 1:**
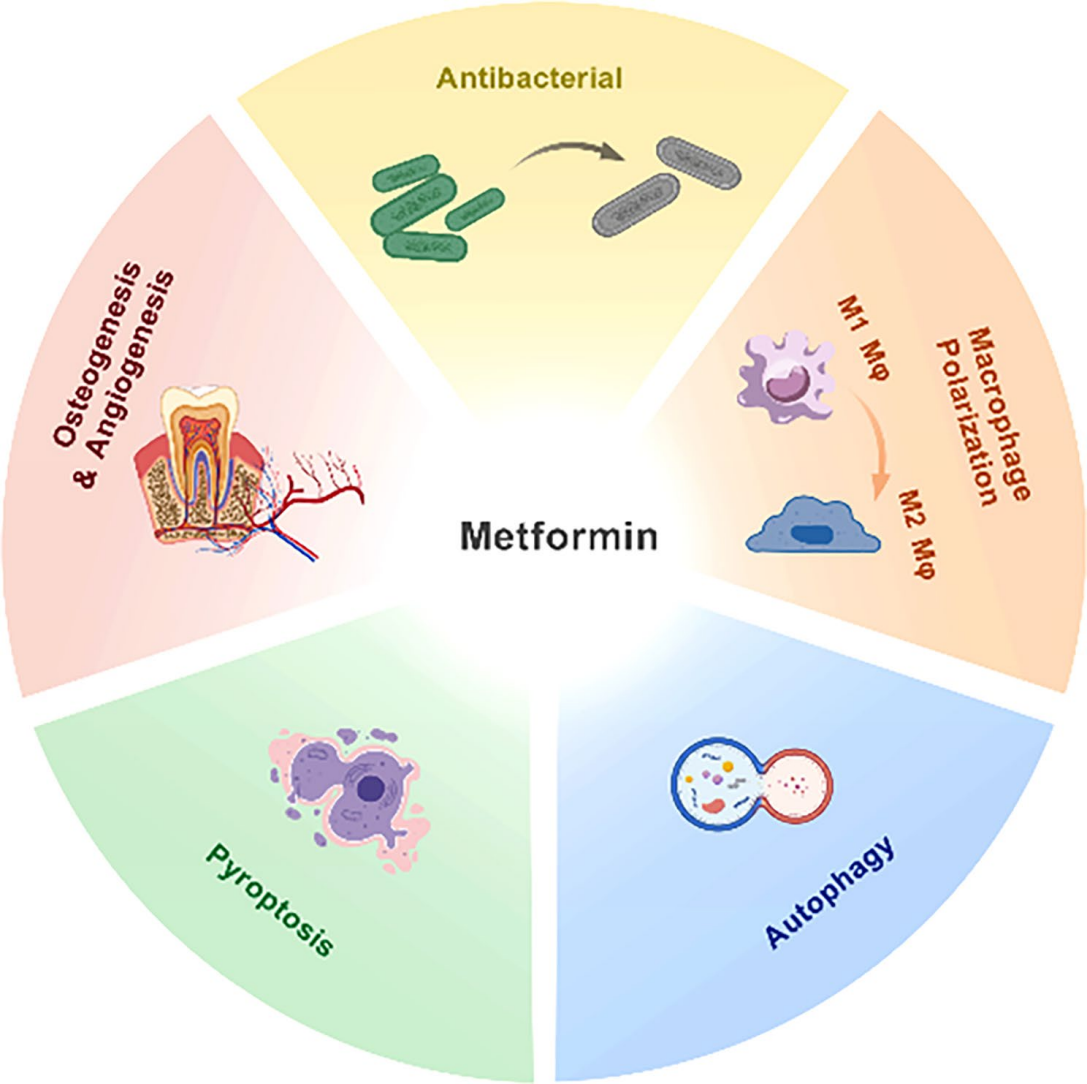
A review of effects of MET in modulating periodontal inflammation

## Materials and methods

This systematic review was conducted in strict accordance with the PRISMA guidelines. The specific procedure is outlined below:

### Initial search

A comprehensive literature search was performed across three core databases: PubMed, Web of Science, and CNKI. The search strategy combined Medical Subject Headings (MeSH) terms and free-text keywords in both Chinese and English. The search utilized predetermined keyword combinations relating to: periodontitis (“Periodontitis”[Mesh], “periodontal disease”, “Periodontitides”, “Pericementitis”), metformin (“Metformin”[Mesh], “Dimethylbiguanidine”, “Dimethylguanylguanidine”, “Glucophage”, “Metformin”, “Hydrochloride”, “Hydrochloride, Metformin”, “Metformin HCl”, “HCl, Metformin”), and broader periodontal terms (“periodontal”), yielding an initial pool of 240 citations.

### Deduplication and screening

All retrieved records were imported into reference management software, where 54 duplicate entries were automatically removed, resulting in 186 unique citations. Two independent reviewers then screened the titles and abstracts of these records, excluding publications that were clearly irrelevant (e.g., those focusing on unrelated conditions or not investigating relevant mechanisms). Any discrepancies were resolved through discussion or consultation with a third reviewer. This process resulted in 79 articles proceeding to full-text assessment.

### Full-text evaluation and final inclusion

The full texts of the 79 potentially eligible articles were retrieved and thoroughly evaluated against the predefined inclusion criteria. Studies were included if they were original research articles directly investigating the effects of metformin on periodontitis or periodontal cells, with content relevant to mechanisms such as antibacterial effects, anti-inflammatory properties, osteogenic differentiation, autophagy, or pyroptosis. Ultimately, 42 studies were included in this review based on their scientific rigor and high relevance to the research topic.

**Fig. 2:**
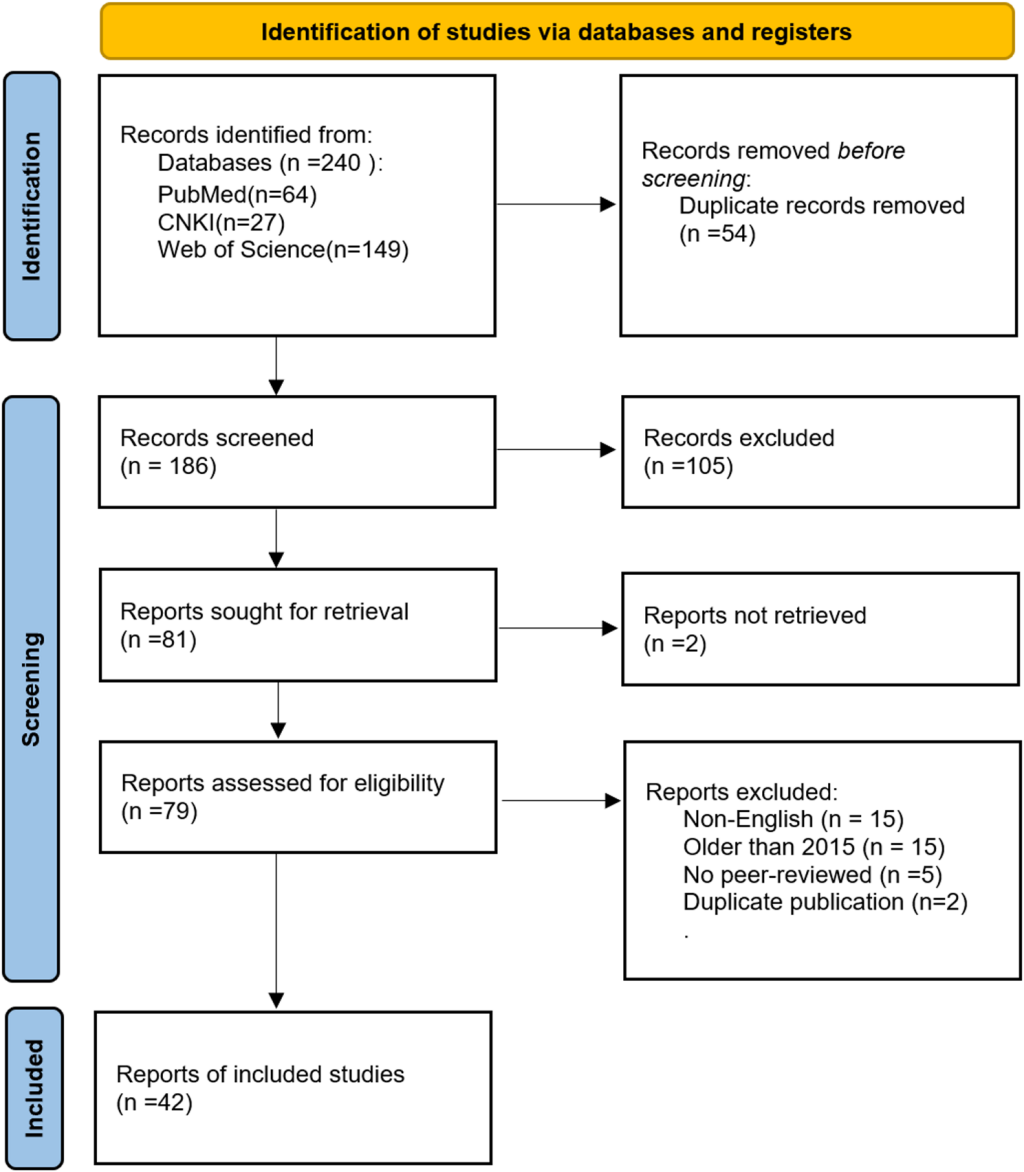
Materials and methods according to the Preferred Reporting Items for Systematic Reviews and Meta-Analyses (PRISMA) guideline [64]

The complete flow of information from initial identification to final inclusion is presented in the PRISMA flow diagram (Fig. 2).

## Effect of MET on periodontal pathogenic bacteria

Contemporary understanding of periodontitis pathogenesis highlights that this inflammatory condition is initiated and maintained by a dysbiotic polymicrobial community, which engages in a complex reciprocal relationship with the host immune response. This interaction creates a self-perpetuating feedback loop [7].

Previous studies have demonstrated that the primary target of MET is Complex I of the electron transport chain [60]. As the first energy-transducing complex in the respiratory chain, Complex I generate the proton motive force required for energy-consuming pathways. Homologs of Complex I are present in the respiratory chains of numerous bacterial species [61].

The inhibition of Complex I by MET leads to reduced ATP production and consequent elevation of AMP levels. This activates the energy sensor AMP-activated protein kinase (AMPK), which enhances phosphorylation of CLIP170 [62], a protein essential for microtubule dynamics. Ultimately, this signaling cascade potentiates the bactericidal capacity of both neutrophils and macrophages [63].

**Fig. 3:**
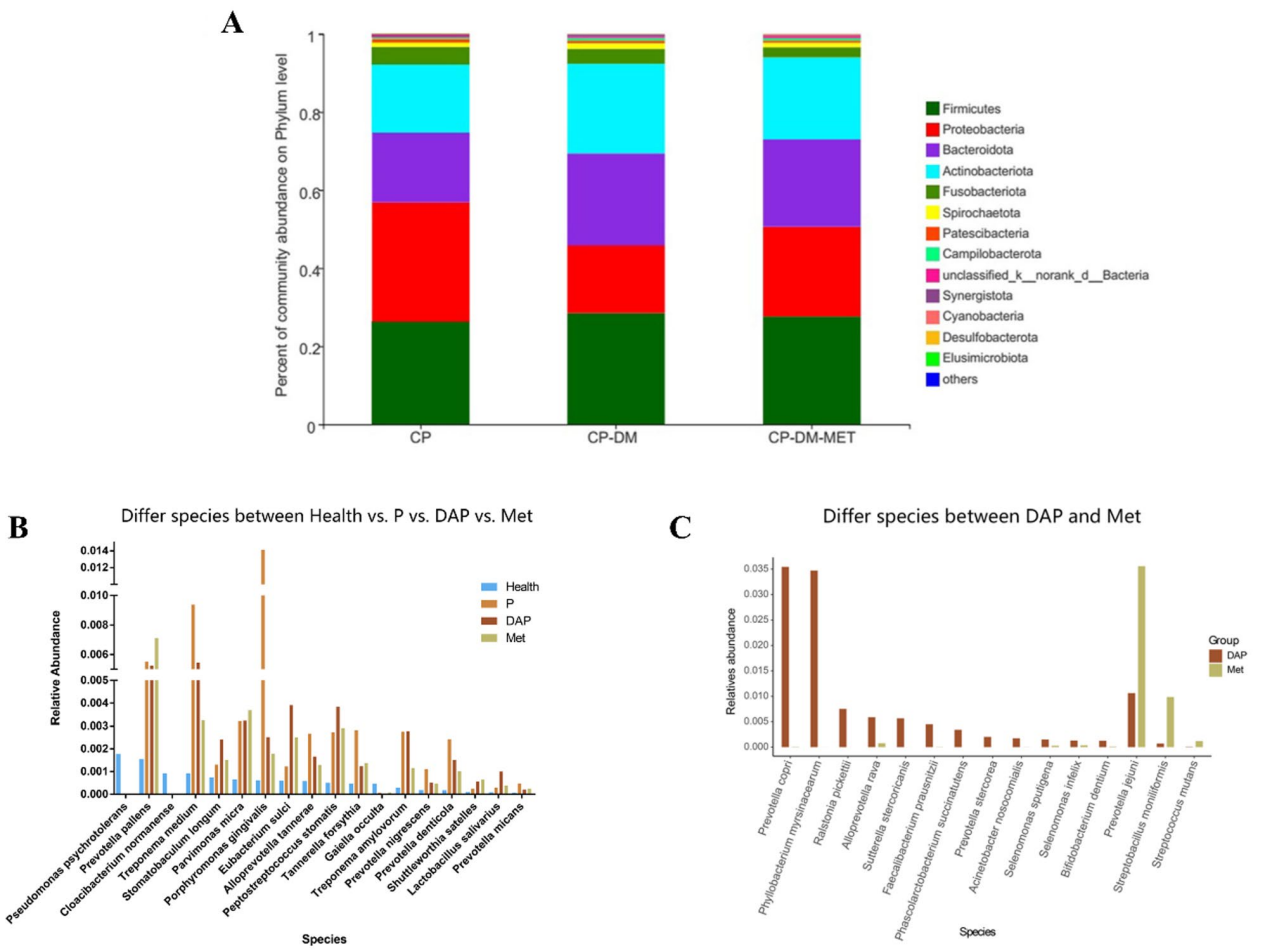
The anti-bacteria effect of MET: **A** Relative abundance of salivary microbiota at the phylum level of the CP, CP-DM, and CP-DM-MET groups. Reproduced with permission from Ref.λ[10]. **B** Bar chart comparing the relative abundance of microbial communities in periodontitis patients (P), periodontitis patients with T2DM (DAP), and DAP patients treated with MET, and Health groups. **C** The relative abundance of salivary microbes in DAP and Met groups. Reproduced with permission from Ref. [8]

Similar research has uncovered MET’s capacity to inhibit periodontal pathogens. MET modulate the oral microbiome by reducing the relative abundance of pathogenic species. Comparative analysis revealed a significant reduction in microbial diversity indices in the CP-DM-MET group compared to CP and CP-DM groups (Fig. 3A). This observation suggests that MET administration may mitigate diabetes-induced alterations in salivary microbiome composition, potentially restoring microbial diversity towards a healthier ecological state. Recent investigations have also demonstrated that the microbial profile of CP-DM-MET group exhibits remarkable similarities to healthy controls (Fig. 3BC) [8]. The study by Yang et al. [9] revealed that MET administration in T2DM patients induces significant compositional changes in the salivary microbiota, particularly affecting Corynebacterium, Nocardia, and Lactococcus populations, suggesting a potential mechanism for metformin’s extra-glycemic benefits.

Despite these promising findings, it is important to note that the consistent presence of the red complex in salivary samples across all study groups (CP, CP-DM, and CP-DM-MET). Quantitative assessment of key periodontal pathogens, including Porphyromonas gingivalis, Treponema denticola, and Tannerella forsythia, demonstrated comparable relative abundances among the three groups (*p* > 0.05) [10]. These findings suggest that neither T2DM nor MET administration significantly influences the composition or prevalence of the red complex in the periodontal environment.

## The mechanisms of metformin-mediated anti-inflammatory action

Dental biofilm and its metabolic byproducts, particularly lipopolysaccharides (LPS), trigger the innate immune system’s first line of defense. Neutrophils, as primary effector cells of the innate immune system, undergo activation, recruitment, and chemotaxis. During their antimicrobial defense functions, these immune cells simultaneously release a cascade of proteolytic enzymes and matrix metalloproteinases (MMPs) that contribute to periodontal tissue destruction. Concurrently, they secrete an array of pro-inflammatory cytokines, including IL-1β, TNF-α, IL-23 [12], and prostaglandin E2 (PGE2), which collectively mediate extracellular matrix degradation and stimulate osteoclastic activity, thereby promoting alveolar bone resorption and periodontal tissue breakdown [11]. The adaptive immune response, mediated by T and B cells, produces specific antibodies and effector molecules to provide defense. However, these same antibodies can also exacerbate tissue damage by triggering secondary inflammatory pathways. In diabetic patients with chronic periodontitis, impaired leukocyte chemotaxis compromises the initial defense barrier. Meanwhile exuberant secretion of inflammatory mediators creates a pro-inflammatory milieu that accelerates periodontal destruction [13]. Furthermore, emerging evidence suggests that programmed cell death pathways, including apoptosis and pyroptosis, play regulatory roles in periodontal inflammation and tissue homeostasis.

MET demonstrates potent immunomodulatory properties, effectively regulating the cascade of inflammatory responses described above. Through multiple molecular mechanisms, MET attenuates inflammatory signaling pathways, thereby inhibiting the progression of chronic inflammation and potentially preventing subsequent tissue damage.

### Metformin as a regulator of macrophage polarization

Macrophages serve dual roles in periodontal pathogenesis, functioning as both immune regulators and phagocytic cells. These cells exhibit remarkable plasticity, differentiating into distinct functional phenotypes. The pro-inflammatory M1 and tissue-reparative M2 macrophage phenotypes play crucial yet opposing roles in the destructive and regenerative phases of periodontal disease, respectively [38]. Pathological progression of periodontitis is associated with a phenotypic shift from M2 to M1 macrophages, contributing to sustained inflammation and tissue destruction [14]. Recent studies by ALA et al. [5, 15] have demonstrated that MET modulates macrophage polarization, promoting a shift towards the M2 phenotype. This MET-induced phenotypic reprogramming results in decreased pro-inflammatory cytokine production and attenuated inflammatory responses, suggesting a potential therapeutic mechanism for periodontitis management.

### Metformin-mediated modulation of cellular autophagy

Autophagy, an evolutionarily conserved Type II programmed cell death mechanism, is characterized by the selective elimination of senescent organelles and superfluous macromolecules via lysosomal degradation. This tightly regulated process, orchestrated by autophagy-related genes (ATGs), serves as a critical quality control mechanism. By generating bioenergetic substrates through macromolecule catabolism, the organism maintains cellular homeostasis [16]. Autophagy exhibits a dualistic function in periodontal pathogenesis [21]. Pathogen invasion can hijack the autophagic machinery to facilitate intracellular survival and replication [20]. This heightened autophagic flux is associated with the inflammatory microenvironment characteristic of periodontal disease pathogenesis [17–19]. Conversely, invading periodontal pathogens are recognized by pattern recognition receptors (PRRs) on innate immune cells, triggering immune-inflammatory cascades that activate protective autophagy to help maintain tissue homeostasis [22]. MET modulates autophagic activity through dual regulation of the mechanistic target of rapamycin (mTOR) and AMP-activated protein kinase (AMPK) signaling pathways.

**Fig. 4:**
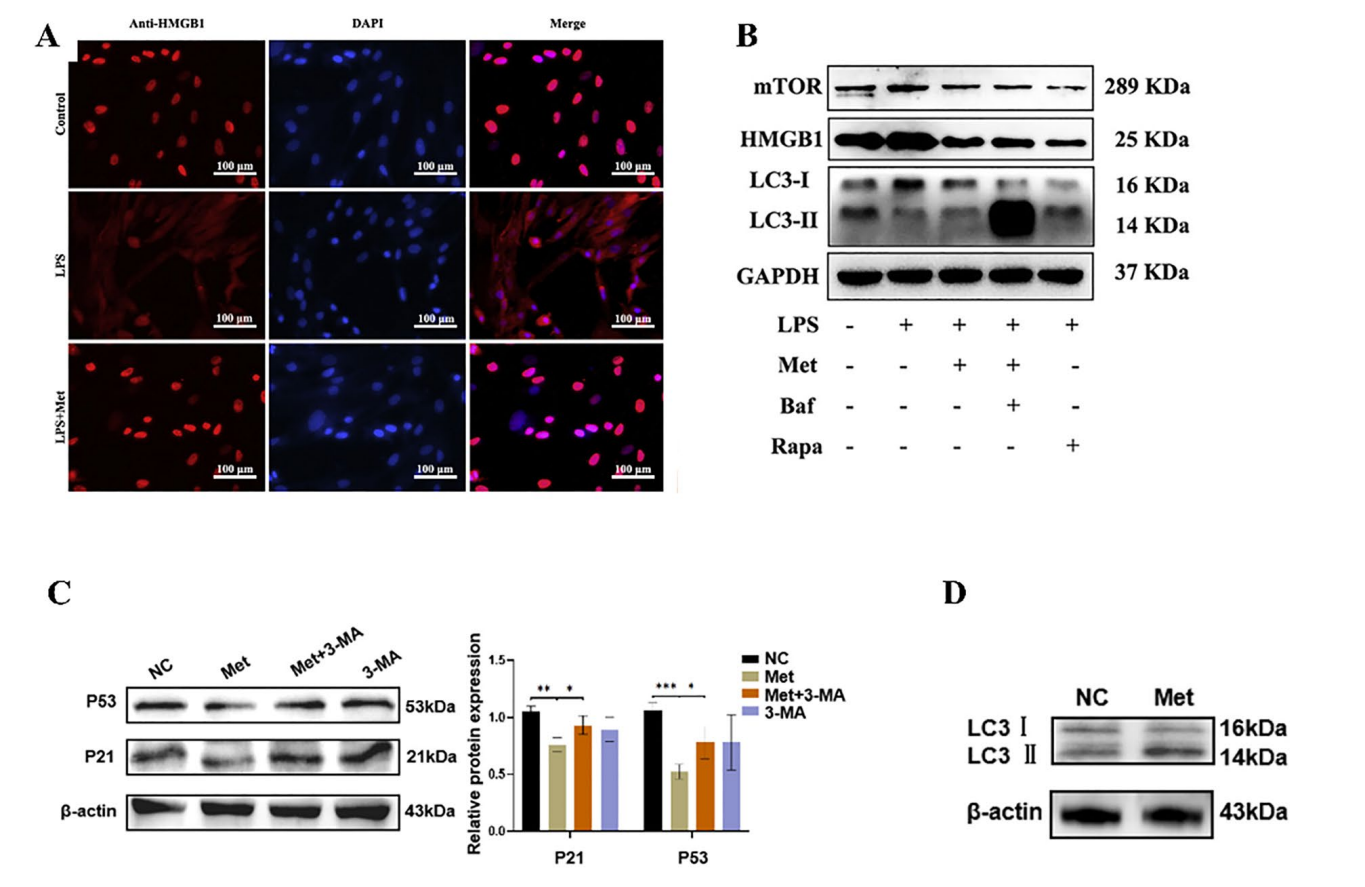
The modulation of autophagy : **A** LPS induced HMGB1 release from the nuclei to the cytoplasm, which was blocked MET. **B** mTOR expression was up-regulated after LPS treatment, was inhibited after MET treatment, and was restored after adding autophagy inhibitor (Bafilomycin A). Reproduced with permission from Ref. [25]. **C** Autophagy inhibitor (3-MA) treatment caused increased expression of senescence biomarkers (P21 and P53 proteins) **D** Autophagy markers (LC3-II/LC3-I) was increased after MET treatment. Reproduced with permission from Ref. [28]

Notably, Morimoto et al. [23] identified elevated levels of high mobility group protein B1 (HMGB1) in the gingival crevicular fluid of periodontitis patients. HMGB1 functions as a critical autophagy modulator in oxidative stress conditions [24]. This damage-associated molecular pattern activates the mechanistic target of rapamycin (mTOR) signaling pathway, subsequently reducing cellular viability, enhancing pro-inflammatory cytokine production and contributing significantly to periodontal disease pathogenesis. Recent investigations by Sun et al. [25] demonstrated that MET effectively downregulates mTOR signaling pathway activity, thereby attenuating HMGB1-mediated oxidative stress responses (Fig. 4AB). This molecular mechanism contributes to the observed amelioration of experimental periodontitis symptoms. Furthermore, these findings provide compelling evidence supporting HMGB1 as a promising therapeutic target for periodontal disease management.

Moreover, emerging research has demonstrated that MET exerts protective effects against age-related pathological alterations through the enhancement of cellular self-renewal mechanisms, particularly autophagy [26]. These MET-mediated processes have shown significant therapeutic potential in mitigating the progression of various age-associated disorders [27], including neurodegenerative conditions, cardiovascular pathologies and periodontitis. Mechanistically, Ye et al. [28] highlights that MET activates the AMPK/SIRT1 signaling axis, enhancing autophagic flux and suppressing hyperglycemia-induced cellular senescence in junctional epithelial cells. These specialized epithelial cells serve as critical biophysical barriers in periodontal immune defense. Their senescence compromises barrier integrity, resulting in increased epithelial permeability and amplified inflammatory responses. MET exerts cytoprotective effects in hyperglycemic conditions by activating SIRT1-mediated autophagic pathways, which mitigate oxidative stress-induced cellular senescence. Furthermore, MET-induced AMPK activation elevates intracellular NAD+ concentrations, creating a positive feedback loop that enhances SIRT1 activity and reinforces cellular defense mechanisms against metabolic stress (Fig. 4CD). Similarly, Kuang et al. [29] pretreated periodontal ligament stem cells (PDLCs) with MET and found that MET effectively counteracts hydrogen peroxide (H2O2)-induced oxidative damage by reducing reactive oxygen species (ROS) accumulation. MET’s protective effects against oxidative stress-induced cellular senescence are mediated through autophagy activation, as evidenced by the reversal of these effects upon treatment with 3-methyladenine, a specific autophagy inhibitor. Collectively, from an anti-aging perspective, MET’s ability to synchronize barrier defense restoration (via epithelial autophagy) and stem cell revitalization (through PDLCs rejuvenation) represents a paradigm shift in periodontal therapy.

### Metformin-mediated modulation of pyroptotic cell death

Pyroptosis, a form of inflammatory programmed cell death, is characterized by the activation of the NOD-like receptor family pyrin domain containing 3 (NLRP3) inflammasome in response to pathogen-associated molecular patterns (PAMPs) such as LPS [30]. This process is mediated by inflammatory caspases (primarily caspase-1) and executed through the cleavage of gasdermin family proteins (GSDMs) [31], resulting in the formation of plasma membrane pores [32]. The pores disrupt cellular osmotic homeostasis, resulting in cell lysis and the subsequent release of pro-inflammatory cytokines. In periodontitis patients, the key components of the inflammasome pathway, including NLRP3, caspase-1, caspase-4, and IL-18, demonstrate markedly increased expression [33]. Pyroptosis drives periodontal destruction via dual mechanisms: the direct loss of HGECs and PDLFs, and the indirect aggravation of damage wherein intracellular contents from P. g.-induced macrophage pyroptosis promote a self-sustaining cycle of bacterial proliferation and inflammation [34]. Moreover, In the context of diabetes-associated periodontitis, hyperglycemic conditions significantly upregulate NLRP3 inflammasome expression and activation.

MET has been shown to effectively suppress both the expression and activation of the NLRP3 inflammasome. This inhibition attenuates pyroptosis-mediated tissue destruction. Tan et al. [37] demonstrated that MET effectively suppresses LPS-induced activation of the NLRP3 inflammasome in PDLCs. The inhibition coincided with a ∼50% reduction in the secretion of pro-inflammatory cytokines IL-1β and IL-18. Complementing this finding, Hu et al. conducted in vitro studies using PDLCs and confirmed that MET exerts its anti-inflammatory effects through dual mechanisms: activation of AMPK signaling and inhibition of mitochondrial complex I activity. These actions collectively suppress NLRP3 inflammasome activation and reduce caspase-1 expression, providing molecular insights into MET’s protective role in periodontal inflammation.

**Fig. 5:**
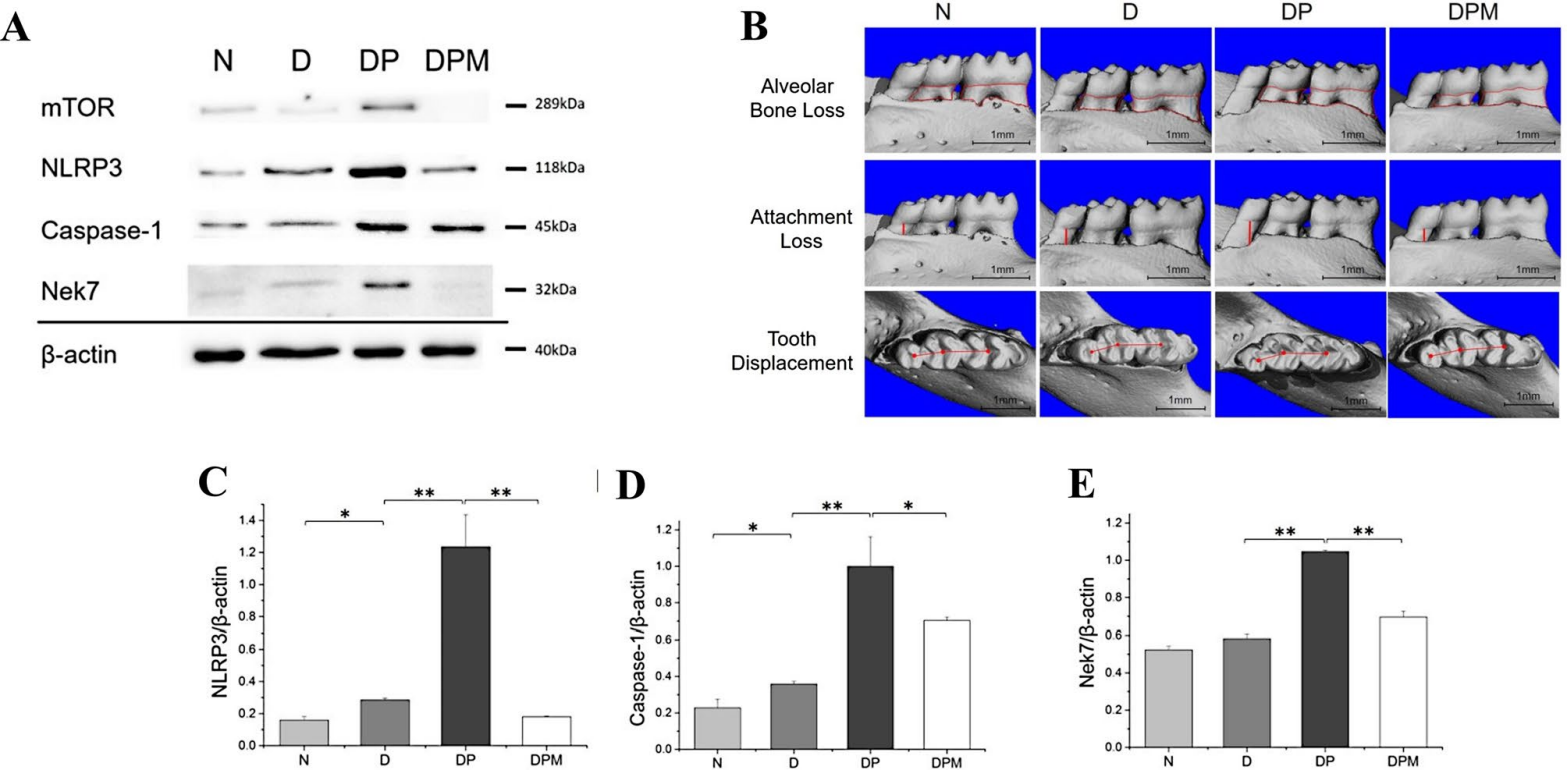
The modulation of Pyroptosis : **A** Decrease expression of mTOR, NLRP3(**C**), caspase-1(**D**) and Nek7(**E**) in the gingival tissue with MET. **B** Three-dimensional reconstructions of the mandibular alveolar bones. Reproduced with permission from Ref. [36]

Through a comprehensive approach encompassing in vitro cellular assays, animal models, and preliminary human observational studies, Zhou et al. [35] investigated MET impact on NLRP3 inflammasome signaling. Their findings demonstrate that MET attenuates NLRP3-mediated pyroptosis by downregulating NEK7 protein expression. Notably, their investigations also revealed that MET maintains its capacity to downregulate the Nek7/NLRP3 axis even in the presence of rapamycin, a specific mTOR inhibitor (Fig. 5). This finding demonstrates that MET’s modulation of Nek7 expression occurs through mTOR-independent mechanisms [36]. The identification of Nek7-mediated pathways not only elucidates a novel mechanism underlying MET’s anti-inflammatory effects but also establishes Nek7 as a promising therapeutic target for the development of innovative treatment strategies.

## Metformin-mediated modulation of osteogenesis: mechanisms and therapeutic applications

The release of LPS from dental biofilm triggers immune cells and osteoblasts to produce inflammatory mediators. This inflammatory cascade activates macrophages and fibroblasts to secrete prostaglandin E2 (PGE2), which promotes osteoclastogenesis and subsequent alveolar bone resorption [38]. The resulting horizontal and vertical bone loss compromises periodontal support, ultimately leading to tooth mobility. While conventional periodontal therapies, including supragingival scaling, subgingival scaling, and root planing, demonstrate anti-inflammatory effects, they lack the capacity to regenerate lost bone tissue. Consequently, current research in periodontal therapy is increasingly focused on developing regenerative approaches, particularly for alveolar bone reconstruction, to address the limitations of traditional treatment modalities.

### The direct osteogenic effect of MET

MET, primarily recognized as an anti-diabetic medication, has demonstrated promising osteoprotective properties in preclinical and clinical studies. Its potential to enhance bone mineral density has led to its application in osteoporosis management, particularly in diabetic patients who are at increased risk of bone fragility and fractures. MET exerts dual beneficial effects on bone metabolism. Primarily, it enhances bone mineralization and regeneration through improved glycemic control, regulation of glucose metabolism, and promotion of cellular migration, as evidenced by increased expression of bone formation markers including osteocalcin, type I collagen, and OSTERIX [39]. Additionally, as previously discussed, MET’s anti-inflammatory properties contribute to its osteogenic potential by creating a favorable microenvironment for bone formation. Ana et al. [40] investigated the osteogenic effects of MET using two osteoblast-like cell models (UMR106 and MC3T3E1). Their findings demonstrate that MET treatment significantly enhances type I collagen synthesis in both cell lines and stimulates alkaline phosphatase (ALP) activity in MC3T3E1 cells. Furthermore, these studies revealed that MET’s direct osteogenic actions are mediated through extracellular signal-regulated kinase (ERK) pathway activation and upregulation of inducible/endothelial nitric oxide synthase (iNOS/eNOS) expression.

### Metformin in bone tissue engineering: dual modulation of osteogenic differentiation and angiogenic potential

Bone tissue engineering has emerged as a highly promising strategy for bone regeneration. Current research emphasizes the critical importance of coupling osteogenesis with angiogenesis, as successful bone regeneration fundamentally relies on the vascular supply of oxygen and essential nutrients to support new bone formation. MET demonstrates significant potential in tissue engineering applications through its dual regulatory effects on bone and vascular regeneration. Mechanistically, MET promotes osteogenic differentiation, matrix mineralization, and bone defect repair via AMPK pathway activation. Additionally, it enhances angiogenic processes through modulation of the AMPK/mTOR/NLRP3 signaling axis, creating a favorable microenvironment for tissue regeneration [41].

MET has demonstrated remarkable capacity to induce osteogenic differentiation across various stem cell types [42, 43]. Extensive research has documented MET’s ability to enhance both proliferation and osteogenic potential in dental pulp stem cells (DPSCs) [44] and PDLSCs [45]. Wang et al. [46] investigated the osteogenic potential of MET using induced pluripotent stem cell-derived mesenchymal stem cells (iPSC-MSCs). Their findings demonstrate that MET treatment maintains iPSC-MSC viability while significantly enhancing osteogenic differentiation capacity. Specifically, MET potently induced osteogenic differentiation with a 6-fold increase in ALP activity and a significant upregulation of key markers: 4-fold for RUNX2 and a remarkable 20- to 30-fold for osterix (OSX). These findings unequivocally confirm its potent osteoinductive properties. Chinese researchers [41] have engineered an innovative calcium phosphate cement-hydrogel-microfiber composite scaffold system designed for both cellular protection and targeted drug delivery. Experimental results demonstrate that MET-incorporated scaffolds significantly enhance osteogenic performance, as evidenced by elevated expression of osteogenic genes, increased ALP activity, and greater osteogenic matrix deposition compared to MET-free control scaffolds.

**Fig. 6:**
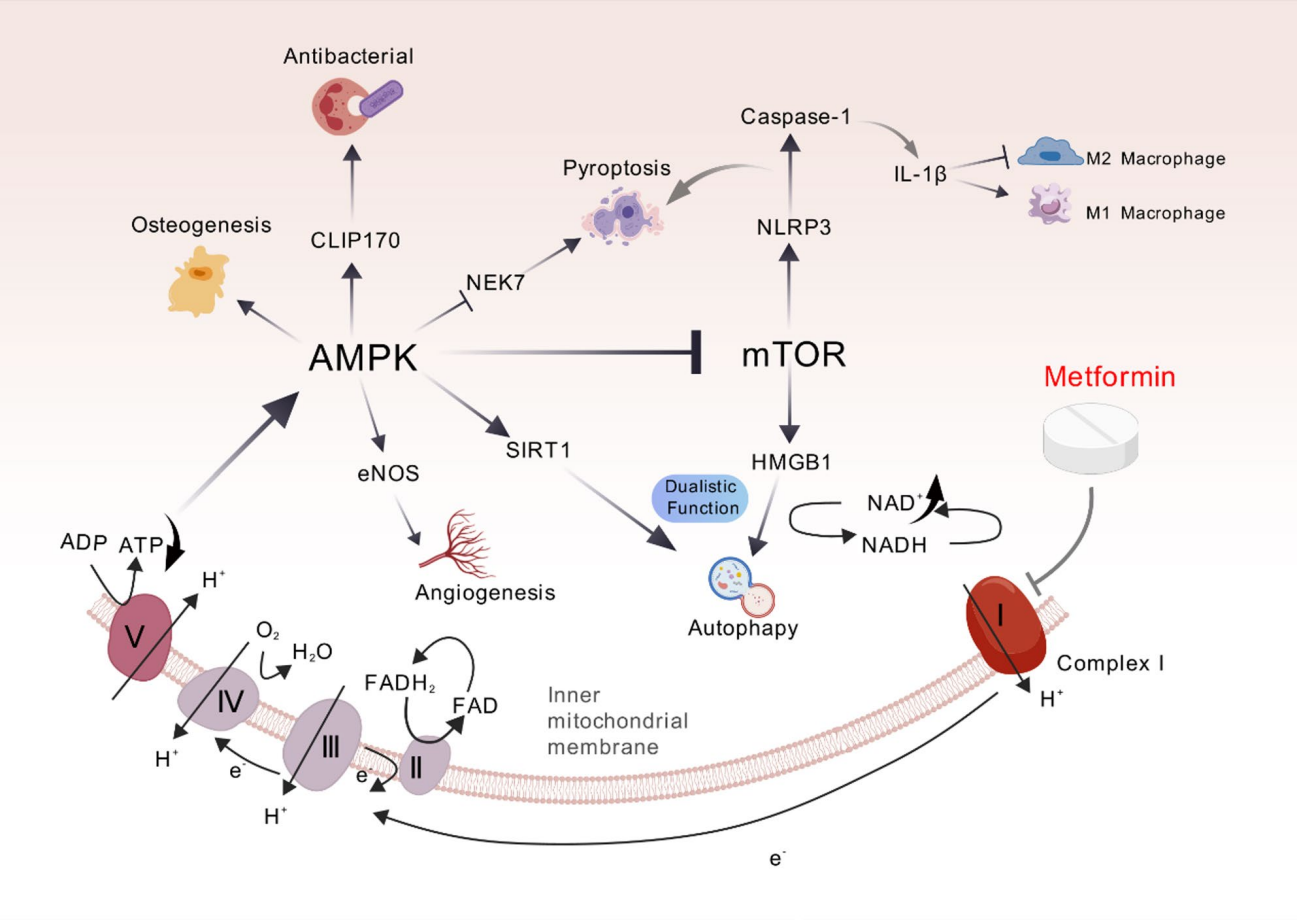
The effects and mechanisms of MET in modulating periodontal inflammation

MET has demonstrated significant angiogenic potential, contributing to enhanced wound healing processes. Tong et al. [47] established an advanced co-culture bone tissue engineering model, revealing that MET treatment significantly upregulates angiogenesis-specific genes, including stem cell factor (SCF) and vascular endothelial growth factor receptor (VEGFR), compared to non-MET controls. This MET-induced molecular environment facilitates umbilical cord-derived mesenchymal stem cell (MSC) migration and promotes vascular network reconstruction.

### The controversy over the optimal therapeutic concentration of metformin

**Fig. 7:**
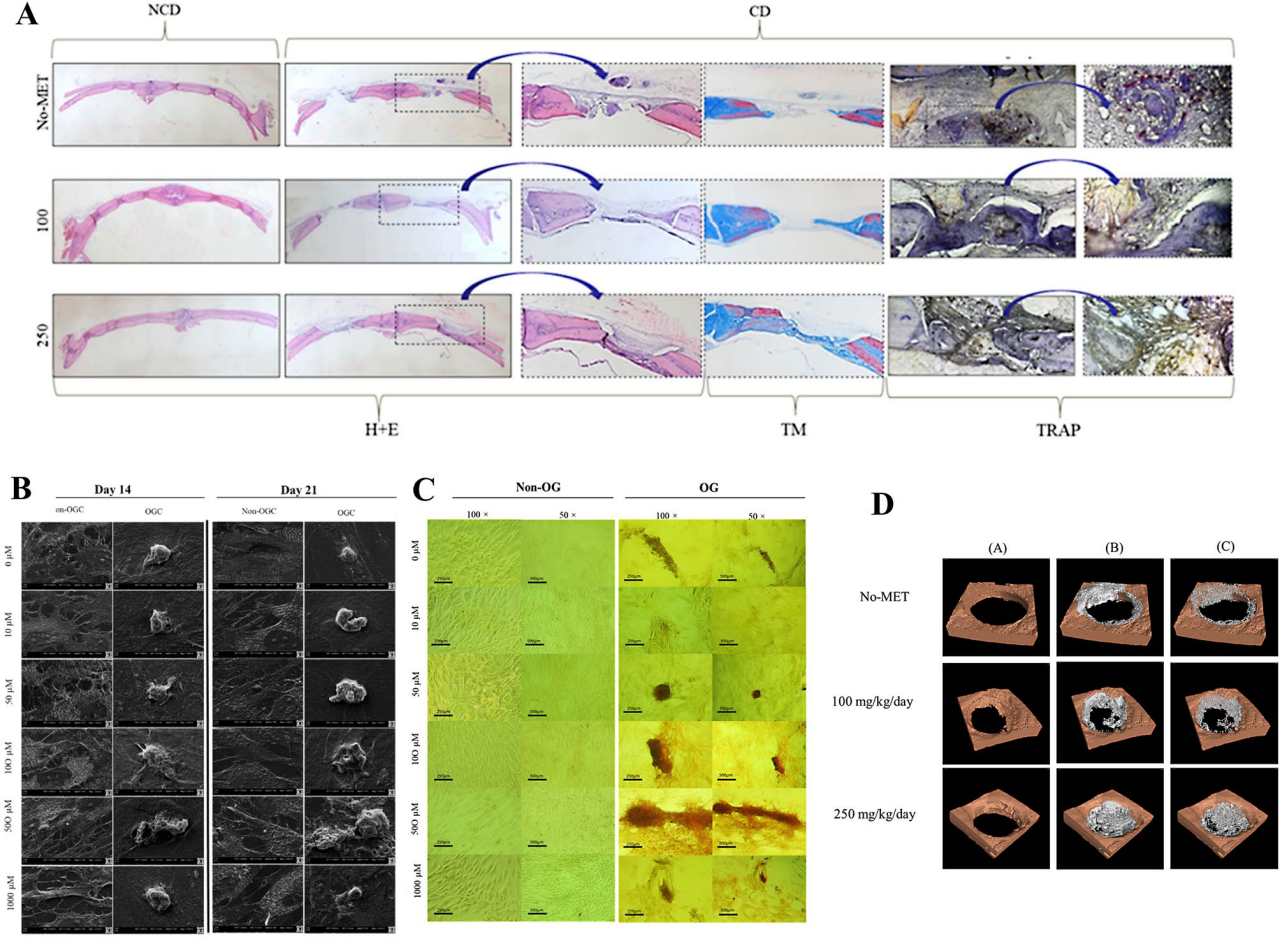
The modulation of osteogenesis: **A-D** shows histology, scanning electron microscopy (SEM) images, micrographs and three-dimensional uCT reconstructions of mineralization at different concentrations. Reproduced with permission from Ref. [49]

In practical applications, the optimal concentration for osteogenesis requires precise determination. Studies [46] have demonstrated that MET enhances osteoblast differentiation via AMPK signaling pathway activation within a concentration range of 0.5–500 µM, with dose-dependent effects on osteogenic marker expression and mineralization capacity. In a rat implantation model, Sun et al. [48] identified 125 µM as the optimal MET concentration. However, concentrations above 200 µM were found to suppress the osteogenic potential of bone marrow-derived mesenchymal stem cells (BMSCs), demonstrating a biphasic dose-response relationship in bone regeneration applications. Through in vitro studies, international research teams have demonstrated that metformin at 500 µM effectively induces osteogenic differentiation of rat adipose-derived stem cells (rASCs) by significantly activating AMPK. Complementary in vivo investigations revealed that daily administration of metformin at 250 mg/kg enhances fracture healing [49]. Recent studies have documented that MET at 100 µM concentration significantly enhances osteogenic differentiation of PDLSCs, while concurrently inhibiting adipogenic differentiation [50] (Fig. 7).

In conclusion, substantial variability exists in reported optimal MET concentrations across different experimental models and applications (Table 1). Furthermore, the translation of these in vitro findings to human clinical contexts remains limited by insufficient evidence and requires more comprehensive preclinical and clinical validation. Significant discrepancies exist between oral dosage and plasma concentrations. Pharmacokinetic investigations reveal that following oral administration of 0.5–1.5 g, plasma MET levels range from 0.4 to 3 µg/ml within 2–4 h post-administration [51]. These pharmacokinetic variations underscore the necessity for developing more precise drug delivery systems and determining optimal therapeutic concentrations to ensure consistent clinical efficacy.

**Table 1:** Effects of MET at different concentrations

Stem cell	Metformin concentration	In vivo / In vitro	Effects
hASCs [49]	1 mmol 250 mg/kg/day	In vitro and In vivo	Enhanced the metabolic and proliferative activity of hASCs, increased the formation of mineralized extracellular matrix, enhanced fracture healing.
hEnSCs [43]	10 wt%	In vivo and In vitro	Be used as a material for guided bone tissue regeneration (GBR), concentrations above 15% inhibit cellular activity.
BMSC [39]	200 mg/kg/day	In vivo and In vitro	Promoted the differentiation of BMSC to osteoblasts, prevented glucocorticoid-induced osteoporosis in rats.
hPDLSCs [44]	50 µg	In vitro	Enhance osteogenic differentiation and mineralization.
BMSC [48]	125µM Over 200µM	In vivo	Optimal MET concentration. Suppressed the osteogenic potential.
iPSC-MSCs [46]	10 µM	In vitro	Enhances osteoblast differentiation

## Advanced MET-incorporated drug delivery systems: a novel therapeutic strategy for periodontitis management

Pharmacological management of periodontitis encompasses both systemic and localized drug delivery approaches. While systemic administration facilitates drug distribution to inaccessible areas such as deep periodontal pockets, it is often limited by suboptimal drug concentrations at the target site and potential systemic adverse effects.

In contrast, localized delivery systems, particularly sustained- and controlled-release formulations, offer significant advantages including higher local drug concentrations, prolonged therapeutic effects, and reduced dosing frequency [53]. However, these localized approaches may present challenges such as complex application procedures [52] and increased treatment time. Substantial clinical evidence has been documented regarding the efficacy of controlled-release drug delivery systems in periodontal pockets (Tables 2 and 3).

**Fig. 8:**
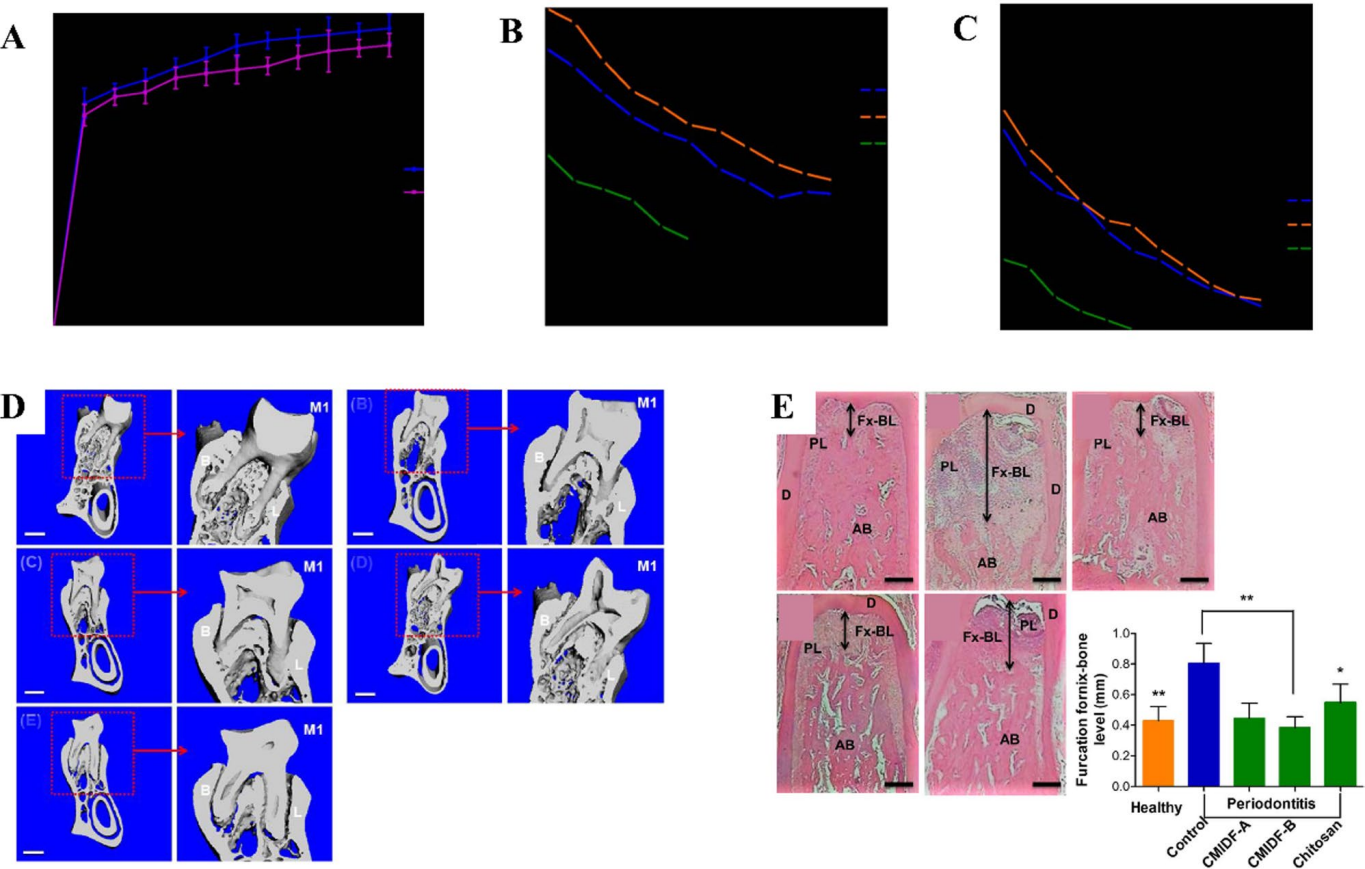
A novel biodegradable chitosan-based MET intrapocket dental film (CMIDF). **A** MET release from CMIDF in a biphasic manner. **B **& **C** shows the inhibition zone of P. gingivalis and T. forsythia growth using CMIDF-A or chitosan film. **D** Micro-CT images of the mandibular first molar and alveolar bone after CMIDF and chitosan treatment. **E** Histological images of interradicular alveolar bone architecture in Reproduced with permission from Ref. [54]

Notably, MET conventionally utilized as an oral hypoglycemic agent, has emerged as a promising therapeutic candidate when integrated with innovative drug carrier systems. Deepak et al. [54] developed an innovative chitosan-based film incorporating MET, which demonstrated significant antimicrobial activity against periodontal pathogens, reduced pro-inflammatory cytokine levels, and promoted alveolar bone regeneration in experimental periodontitis models (Fig. 8). Pradeep et al. [55] investigated the adjunctive use of 1% MET gel following scaling and root planing (SRP) procedures. Their clinical findings demonstrated superior clinical attachment level (CAL) gain, achieving a 30% reduction in intrabony defect (IBD) depth—a 15-fold greater improvement over the control. Some researchers have developed an innovative bilayer film system through a double-casting and pressurization technique. The inner drug-loaded layer, composed of 6% sodium carboxymethyl cellulose (CMC), enables controlled release of MET, while the outer adhesive layer, fabricated from sodium thioglycolate alginate, enhances periodontal pocket retention. This engineered film exhibits optimal physical properties, including uniform thickness, mechanical strength, and flexibility, facilitating easy insertion and prolonged retention in periodontal pockets [56]. Furthermore, innovative approaches have explored the synergistic combination of platelet-rich fibrin (PRF) and MET for enhanced periodontal regeneration [57]. These therapeutic approaches collectively demonstrate significant potential in mitigating periodontal tissue destruction and offering superior regenerative outcomes compared to individual therapies.

**Table 2:** Randomized clinical trials of metformin in periodontitis treatment

Author	Number of patients	Male-to-Female ratio	Age mean	Intervention		Follow-up	Outcomes
				Test	Control		
Pankaj [65]	90	44:46	34.32 ± 5.09	SRP + 1.2% RSV gel (30), SRP + 1%MF gel (30)	SRP+ placebo gel (30)	0,6,12months	Clinical and radiographic parameters were significantly better in test groups than control
Pradeep [55]	65	38:27	32.4 ± 2.1	1%MF (33)	SRP + 0%MF (32)	0,3,6months	Clinical and radiographic parameters were significantly better in test groups than control
Pradeep [57]	136	68:68	41	OFD + PRF (34) OFD + 1% MF (34) OFD + PRF+1% MF (34)	OFD (34)	0,9months	Clinical and radiographic parameters were significantly better in test groups than control, OFD + PRF+1% MF produced the strongest effect
Patil [66]	15	6:9	-	SRP+curettage+intrapocket application of 1.5% MF gel (15 sites)	SRP+curettage+intrapocket application of placebo gel (15 sites)	0,3,6months	Clinical and radiographic parameters were significantly better in test groups than control

MF, metformin gel; PRF, platelet-rich fibrin; OFD, open-flap debridement; SRP, scaling and root planning

In the management of periodontal intrabony defects (PIDs), advanced drug-delivery bone tissue engineering systems also demonstrate significant regenerative potential. Fang et al. [58] engineered a metformin-loaded scaffold gelatin/nano-hydroxyapatite/metformin (GHMS) for periodontal bone regeneration. The MET-loaded scaffold system tuned the periodontal regenerative niche through a concerted modulation of osteoblast-osteoclast crosstalk and inflammatory microenvironment remodeling. This targeted mechanism translated into a significantly enhanced osseous phenotype, characterized by a BV/TV of 70% and a minimal fibrous tissue formation. International researchers [59] engineered metformin MET-functionalized sol-gel derived silica/zirconia (SiO₂/ZrO₂) coatings to modulate mesenchymal stem cell (MSC) behavior. Adipose-derived human MSCs cultured on these MET-modified surfaces demonstrated sustained fibroblast-like morphology with extended cytoskeletal projections, enhanced calcium-phosphate deposition, and significant elevation of osteocalcin (OCN) and osteopontin (OPN) secretion. Beyond its role as a scaffold-loaded therapeutic agent, MET also demonstrates unique material-modulating capabilities. Specifically, in polydopamine-templated hydroxyapatite (tHA) scaffolds, MET reduces oxidative stress generated by the scaffolds and improves the biocompatibility.

**Table 3:** Novel therapeutic strategies for Metformin

Combination	Metformin concentrations	Advantages and Effects
Degradable alginate-fibrin hydrogel fibers (encapsulate and deliver metformin and periodontal ligament stem cells) [67]	50 µM	Alginate-fibrin hydrogel fibers excel in biocompatibility and injectability for minimally invasive delivery. They provide localized, sustained release of metformin and continuous induction of hPDLSCs, enabling effective regeneration of maxillofacial bone and periodontal tissues.
Nanocarrier-Assisted Delivery of Metformin (Met@HALL) [68]	50 µM	Met@HALL enables sustained, localized metformin delivery via a green fabrication process. It enhances PDLSC adhesion, proliferation, and osteogenesis under high glucose by mitigating oxidative stress and rescuing cell cycle arrest. In diabetic rats, it accelerates bone turnover, reduces inflammation, and promotes angiogenesis, ultimately regenerating periodontal tissue through improved ER homeostasis and stem cell function.
Met@TA-ZIF-PSG patch [69]	2.5 wt%	Compared to traditional periodontal biomaterials, this patch offers superior mechanical flexibility and sustained drug release, courtesy of its ultralong PDA-mSF and TA-ZIF components. It also exhibits excellent tissue affinity, antioxidant, anti-inflammatory, and anti-aging properties, which are conferred by PDA, TA, and the Met-ZIF system. More importantly, it promotes M2 macrophage polarization, leading to immunomodulation and osteogenesis. Through these synergistic effects, the patch achieves alveolar bone regeneration and orderly PDL fiber arrangement, presenting a promising strategy for treating diabetic periodontitis.
Metformin carbon dots-based osteogenic and protein delivery system (MCDs) [70]	0.2 mg/mL	The Gel/MCDs@IGF-1 composite hydrogel is a biocompatible system that sequentially releases MCDs and IGF-1. It rapidly releases MCDs to exert anti-inflammatory effects and activate the PI3K/AKT pathway for osteogenesis, followed by sustained IGF-1 delivery to recruit stem cells and support regeneration. Demonstrating efficacy in vitro and in vivo, this degradable, off-the-shelf system significantly promotes alveolar bone repair in periodontitis.
Tetrahedral Framework Nucleic Acid (tFNA)-Loaded Metformin Complex (TMC) [71]	150µM/250 mg·kg⁻¹	The tFNA-based TMC system efficiently delivers metformin with high stability and bioavailability. It potently inhibits inflammation and NLRP3-mediated pyroptosis via the AMPK/NF-κB pathway, while significantly alleviating alveolar bone loss in diabetic periodontitis. This programmable nanocarrier offers prolonged periodontal protection, showcasing superior therapeutic efficacy.

## Summary and prospects

This article reviews the effects and mechanisms of MET on periodontitis. While primarily recognized as a first-line antidiabetic agent, MET demonstrates significant pleiotropic effects in periodontal therapy. Recent research has focused on developing innovative MET-based delivery systems, marking a significant advancement in periodontitis therapy. These approaches include localized delivery platforms and tissue engineering integration, involving injectable hydrogels, electrospun nanofibers and MET- loaded scaffolds.

Despite promising advancements, several critical knowledge gaps persist in MET-based periodontitis therapy:

1. **Mechanistic understanding**:

- Incomplete elucidation of AMPK-independent pathways.
- Limited data on MET’s immunomodulatory effects in periodontal microenvironment.

2) **Dosage optimization**:

- Unclear therapeutic window for alveolar bone regeneration (current range: 50–500 µM).
- Insufficient pharmacokinetic data in periodontal tissues.

3) **Clinical translation**:

- Limited large-scale clinical trials for novel delivery systems.
- Lack of standardized protocols for combination therapies.

Future research directions should prioritize:

- Systematic investigation of MET’s molecular targets beyond AMPK.
- Dose-response studies in relevant animal models.
- Randomized controlled trials evaluating long-term efficacy and safety.
- Development of standardized evaluation metrics for regenerative outcomes.

In conclusion, comprehensive periodontitis management requires a multifaceted approach encompassing: microbial control, inflammation modulation and tissue regeneration. MET demonstrates remarkable therapeutic potential across all these critical aspects, as a promising candidate for next-generation periodontitis therapies, bridging the gap between antimicrobial treatment and regenerative medicine.

## Data Availability

No new data were generated for this review. All data supporting the findings discussed are available from the original publications cited in the reference list.
